# The potential mechanism of HIF-1α and CD147 in the development of triple-negative breast cancer

**DOI:** 10.1097/MD.0000000000038434

**Published:** 2024-06-07

**Authors:** Menghe Chen, Zitao Liu, Kai Zheng, Chaohua Hu, Pai Peng

**Affiliations:** a School of Medicine, Wuhan University of Science and Technology, Wuhan, People’s Republic of China; b Department of Breast and Thyroid Surgery, Xiaogan Central Hospital and Xiaogan Hospital Affiliated to Wuhan University of Science and Technology, Xiaogan, People’s Republic of China.

**Keywords:** CD147, HIF-1α, immunohistochemistry, survival analysis, triple-negative breast cancer

## Abstract

**Background:**

Triple-negative breast cancer (TNBC) is a subtype of breast cancer with a poor prognosis, and the outcomes of common therapy were not favorable.

**Methods:**

The samples of 84 patients with TNBC and 40 patients with breast fibroadenoma were collected in the pathology department specimen library of our hospital. The prognosis of patients was obtained through outpatient follow-up information, telephone and WeChat contacts, and medical records. The mRNA expression was analyzed using bioinformation and quantitative real-time polymerase chain reaction (qPCR). The protein expression was determined by hematoxylin-eosin staining and immunohistochemical staining. The results of survival analysis were visualized using Kaplan–Meier curves.

**Results:**

The immunohistochemical staining showed that hypoxia-inducible factor-1alpha (HIF-1α) was mainly distributed in the nucleus and cytoplasm, while CD147 is mainly distributed in cell membrane and cytoplasm. The qPCR results exhibited that the expression level of HIF-1α and CD147 in TNBC tissue was significantly higher than that in breast fibroadenoma tissue. The expression of HIF-1α was related to the histological grade and lymph node metastasis in TNBC, and the expression of CD147 was related to Ki-67, histological grade and lymph node metastasis. There was a positive relationship between the expression of CD147 and HIF-1α. The upregulated expression of CD147 was closely related to the poor prognosis of OS in TNBC.

**Conclusion:**

CD147 could be a biomarker for the prognosis of TNBC and closely related to the expression of HIF-1α.

## 1. Introduction

According to the GLOBOCAN database developed by the International Agency for Research on Cancer, the number of cases of breast cancer has surpassed that of lung cancer to become the cancer type with the highest number of cases and the incidence rate of breast cancer also ranks first in this list.^[1]^ At present, the molecular typing including Luminal A type, Luminal B type, human epidermal growth factor receptor 2 over-expression and triple-negative breast cancer (TNBC) was closely related to the prognosis of BC.^[2,3]^ As previously reported, TNBC is one of the highly aggressive types and accounts for 15% to 20% of BC with a poor prognosis.^[4,5]^ Chemotherapy is now the main treatment for TNBC patients, but a higher risk of chemotherapy resistance and more serious side effects were observed in the treatment.^[6]^ Therefore, it is urgent to explore the mechanism of the development of TNBC and find potential therapeutic targets.

Hypoxia-inducible factor-1alpha (HIF-1α) is a transcription factor with an essential role in the response to hypoxia,^[7]^ and it has been reported that the intra-tumoral hypoxia has a more negative role in TNBC than other subtypes of BC.^[8]^ At present, some studies identified that HIF-1α was involved in tumor angiogenesis and metabolic reprogramming, as well as multiple steps in the process of breast cancer invasion and metastasis.^[9]^ Moreover, Fourie et al found that the drug resistance of BC patients can be improved by regulating the expression of HIF-1α,^[10]^ which indicated that HIF-1α may be a promising target for the treatment of TNBC.

CD147, also known as an extracellular matrix metalloproteinase inducer, is a cell-surface glycoprotein and widely expressed in various cancers.^[11]^ The abnormal expression of CD147 was observed in various cancers including BC.^[12]^ Besides, it promotes tumor invasion, growth, and metastasis by stimulating matrix metalloproteinase synthesis in neighboring fibroblasts and inhibiting cell apoptosis.^[13]^ Furthermore, it could interact with some oncogenic proteins resulting in the deterioration of tumor malignancy and drug resistance.^[14]^ However, the function of CD147 in TNBC was not clear.

Thus, in this study, we tried to identify the expression of HIF-1α and CD147 in TNBC and analyzed the prognosis of them.

## 2. Methods

### 2.1. Data collection and processing

We collected the TNBC patient-related case data of the target experimental wax block in the medical record system and searched for the target wax block with complete tissue structure in the pathology department specimen library. The wax block embedding boxes are grouped and coded. The wax on the surface of the wax block tissue is removed by a slicer followed by slicing, with the thickness of each paraffin section being about 3 to 4 um. The paraffin sections are dispersed and placed in the floating pool of the spreading machine at 40°C to 45°C. Then paraffin sections were fished out and adhered to the anti-falling glass slide. After sections were air-dried, they were placed in a constant-temperature blast drying oven for 20 minutes at 60°C to 65°C.

In this study, the patients in the TNBC group were followed up after the operation, and the follow-up time was until June 2022. The follow-up information including disease free survival (DFS) and overall survival (OS) was recorded through outpatient follow-up information, telephone and WeChat contacts, and medical records.

### 2.2. Hematoxylin-eosin staining

The sections were dewaxed in xylene for 10 minutes. Then, these sections were subjected to gradient alcohol hydration (100% for 2 min, 95% for 2 min, 80% for 2 min) and washed for 2 minutes with water. Next, the sections were stained with hematoxylin for 5 minutes and washed for 10 minutes with water. The 0.5% hydrochloric acid alcohol was used to differentiate for 5 seconds. In order to dye hematoxylin blue, the sections were soaked in saturated lithium carbonate for several seconds followed by the water wash for 15 seconds. Then, after the sections were subjected to gradient alcohol dehydration (80% for 2 min, 95% for 2 min, 100% for 2 min), they were soaked in xylene for 2 minutes. Finally, neutral gum was used for sealing sections.

### 2.3. Immunohistochemical staining

The sections were put into xylene for 5 minutes. Then, the samples were subjected to gradient alcohol hydration (100% for 10 min, 90% for 10 min, 70% for 10 min). After samples were washed 3 times with PBS for 3 minutes, they were flicked dry and put into a wet box. Then, hydrogen peroxide (3%) was added in sections for incubation for 15 minutes at room temperature. After sections were washed 3 times with PBS for 3 minutes and flicked dry, they were incubated with 100 μL primary antibodies against HIF-1α or CD147 for 15 minutes in the dark at room temperature. Next, the sections were incubated with 100 μL secondary antibodies for 45 minutes at room temperature after they were washed 3 times with PBS for 3 minutes and flicked dry. The sections were color developed by 100 μL DAB working solution. Sections were counterstained with hematoxylin, differentiated with alcohol hydrochloride (0.1%) and turned back to blue using ammonia (0.5%). The sections were dehydrated by graded ethanol (80% for 2 min, 95% for 2 min, 100% for 2 min) and cleared 3 times for 3 minutes with xylene and coverslipped before being visualized with an Olympus microscope.

### 2.4. Quantitative real-time polymerase chain reaction

Total RNA Extractor was applied to extract total RNA from normal and CRC cells and tissues. After the ratio of A260/A230 and A260/A280 was measurement, reverse transcription of RNA samples and quantitative real-time polymerase chain reaction was performed with SwsScript All-in-One First-strand-cDNA-synthesis SuperMIx for quantitative real-time polymerase chain reaction (qPCR) (One step gDNA Remover) in the 2720 Thermal Cycler. The expressions of CD147 and HIF-1α were determined using the 2−^ΔΔCt^ method with glyceraldehyde-3-phosphate dehydrogenase chosen as control references.

PCR reaction conditions were set in accordance with the manufacturer’s instructions: 60 seconds at 95°C, then 20 seconds at 95°C, 20 seconds at 55°C, and 30 seconds at 72°C for 40 cycles. The primer sequences are shown in Table 1.

**Table 1:** All primers in qPCR experiments in this study.

ID	Forward sequence(5’-3’)	Reverse sequence(5’-3’)
GAPDH	CCCATCACCATCTTCCAGG	CATCACGCCACAGTTTCCC
CD147	GAAGTCGTCAGAACACATCAACG	TTCCGGCGCTTCTCGTAGA
GAPDH	GAACGTCGAAAAGAAAAGTCTCG	CCTTATCAAGATGCGAACTCACA

GAPDH = glyceraldehyde-3-phosphate dehydrogenase.

### 2.5. Statistical analysis

All experimental data in this study were analyzed by SPSS26.0 computer software. The expression of HIF-1α and CD147 in TNBC and breast fibroadenoma, and the relationship between the expression of the 2 indexes and the clinicopathological factors of TNBC patients were analyzed by 2 tests (Fisher exact test was used when the theoretical value was less than 5). The correlation between the 2 indexes was analyzed by the Spearman correlation test. The relationship between the expression of the 2 indexes and the prognosis of patients was analyzed by Kaplan–Meier, and the Log-rank test of survival analysis was carried out. Using the database of breath cancer gene-expression miner v4.9 (BC-genexminer v4.9), 1988 TNBCs with complete HIF-1α information were screened from 10,923 cases of breast cancer and 2022 TNBCs with complete CD147 information were screened from 10,923 cases of breast cancer, and the relevant survival analysis curves (DFS, OS) were obtained through Kaplan–Meier. Cox multivariate analysis was used to analyze the expression of the 2 indexes, the clinicopathological risk factors and prognosis of TNBC patients. All p values were tested by statistical bilateral test, and *P* < .05 indicated that the difference was statistically significant.

## 3. Results

### 3.1. The characteristics of the participants

In this study, a total of 84 TNBC patients were enrolled. The baseline characteristics of patients with TNBC were shown in Table 2. There were fewer young patients than the old patients (30 [35.7%] vs 54 [64.3%]). The histological grade of the I + II group was more than that of the III group (62 [73.8%] vs 22 [26.2%]). Furthermore, the patients with high expression of Ki-67 were more than the patients with low expression of Ki-67 (60 [71.4%] vs 24 [28.6%]). Besides, there were more patients with lymph node metastasis than patients without lymph node metastasis (58 [69%] vs 26 [31%]).

**Table 2:** The clinical characteristics of patients.

Variables	Group	Numbers	Ratio (%)
Age	<45	30	35.7
	≥45	54	64.3
Tumor size	<2 cm	42	50.0
	≥2 cm	42	50.0
Vascular tumor thrombus	Yes	48	57.1
	No	36	42.9
Histological grade	I + II	62	73.8
	III	22	26.2
Ki-67	High	60	71.4
	Low	24	28.6
Lymph node metastasis	Yes	58	69.0
	No	26	31.0

Histological grading All sections were graded histologically by 2 senior pathologists according to the latest Nottingham grading standard. Ki-67 ≥ 20 was considered as high expression.

### 3.2. The prognosis of hypoxia-inducible factor-1alpha and CD147 in triple-negative breast cancer using bioinformation

**Figure 1.**
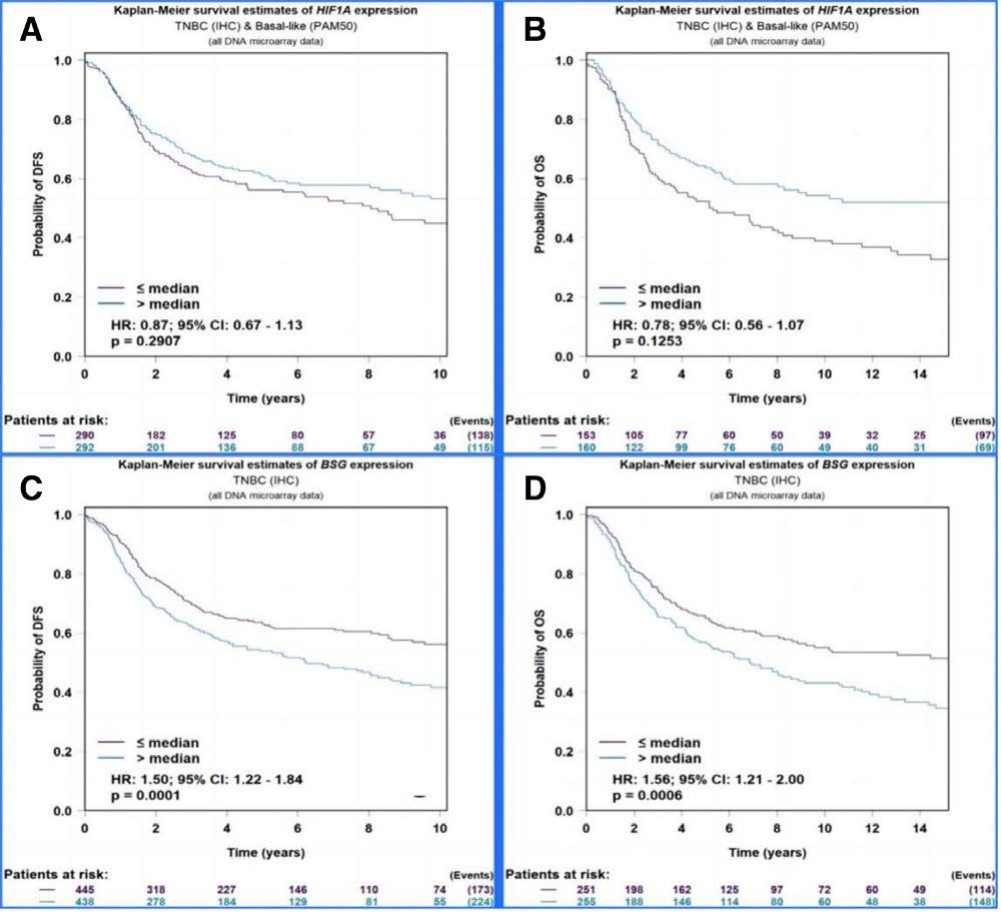
The correlation between the expression of HIF-1α and CD147 and the prognosis of TNBC. (A) The correlation between the expression of HIF-1α and DFS. (B) The correlation between the expression of HIF-1α and OS. (C) The correlation between the expression of CD147 and DFS. (D) The correlation between the expression of CD147 and OS. DFS = disease free survival, OS = overall survival, TNBC = triple-negative breast cancer.

Using the database of breath cancer gene-expression miner v4.9 (BC-genexminer v4.9), 1988 TNBCs with complete HIF-1α information and 2022 TNBCs with complete CD147 information were screened from 10,923 cases of BC. The Kaplan–Meier curves exhibited that there was no significant correlation between the expression of HIF-1α and the prognosis of TNBC (DFS: *P* = .290 and OS: *P* = .125) (Fig. 1A-B). The expression of CD147 was significantly correlated with the prognosis of TNBC (DFS: *P* < .001 and OS: *P* < .001) (Fig. 1C-D).

### 3.3. The prognosis factors in triple-negative breast cancer

Then, we tried to explore the function of HIF-1α, CD147 and clinical characteristics in the TNBC using multivariate Cox analysis. The results showed that only CD147 could independently predict the prognosis of TNBC (*P* < .05) (Table 3).

**Table 3:** Multivariate Cox analysis of prognostic factors in 84 patients with TNBC.

Variables	HR	95% CI	*P*
HIF-1α	1.401	0.565–3.475	.467
CD147	2.671	1.890–8.013	.030
Tumor size	1.026	0.508–2.073	2.073
Lymph node metastasis	0.523	0.231–1.1.82	1.182
Histological grade	1.050	0.495–2.228	2.228
Ki-67 expression	1.473	0.645–3.364	3.364
Vascular tumor thrombus	0.595	0.303–1.171	1.171
Age	0.825	0.413–1.648	1.648

CI = confidence interval, HIF-1α = hypoxia-inducible factor-1alpha, HR = hazard ratio.

### 3.4. The expression of hypoxia-inducible factor-1alpha in breast cancer

**Figure 2.**
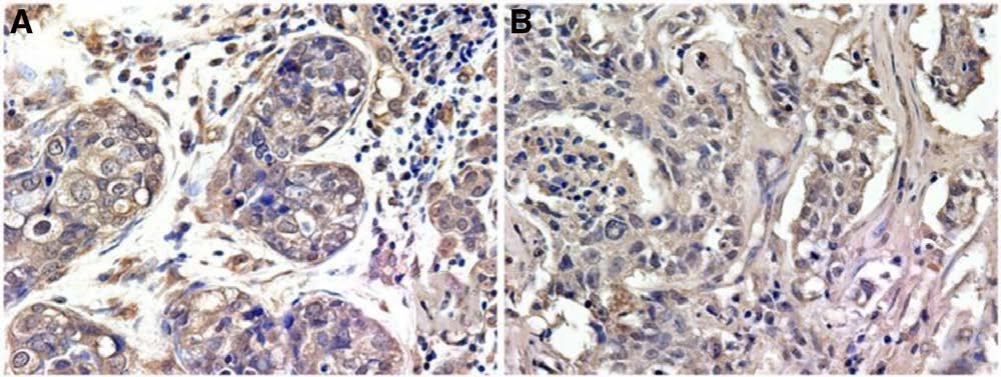
The positive expression characteristics of HIF-1α in the TNBC and fibroadenoma of the breast. (A) The expression of HIF-1α in TNBC. (B) The expression of HIF-1α in fibroadenoma of the breast. HIF-1α = hypoxia-inducible factor-1alpha, TNBC = triple-negative breast cancer.

**Figure 3.**
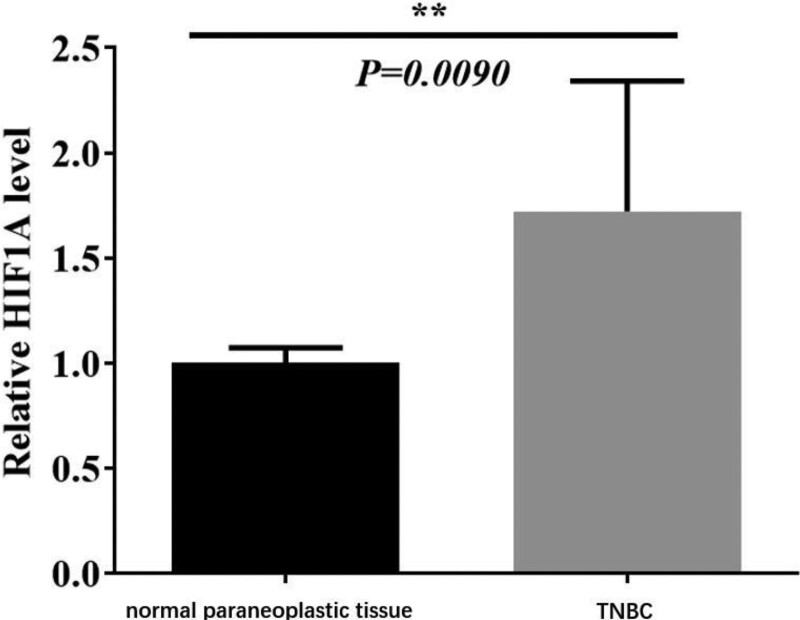
The expression of HIF-1α in TNBC and normal paraneoplastic tissue. **P < .01. HIF-1α = hypoxia-inducible factor-1alpha, TNBC = triple-negative breast cancer.

Next, we identified the expression of HIF-1α in different types of breast cancer. From Table 4, it was obvious that 63 patients had positive expression of HIF-1α among 84 TNBC patients, while 11 patients had positive HIF-1α expression among 40 patients with fibroadenoma of the breast. The further experiments showed that HIF-1α was expressed in the nucleus and cytoplasm which exhibited pale yellow and brownish yellow granules, and a few cells were strongly stained with dark brownish yellow (Fig. 2A-B). Besides, the qPCR results identified that expression of HIF-1α was significantly upregulated in TNBC samples compared with normal samples (Fig. 3, *P* < .01).

**Table 4:** The positive rate of HIF-1α expression in TNBC and fibroadenoma of breast.

Group	Number	HIF-1α positive	χ^2^	*P*
TNBC samples	84	63 (75.0%)	25.407	<.001
Fibroadenoma of breast	40	11 (27.5%)		

TNBC = triple-negative breast cancer.

### 3.5. The correlation between hypoxia-inducible factor-1alpha expression and clinical characteristics in triple-negative breast cancer

As shown in Table 5, it can be seen that HIF-1α was positively expressed in 43 (49.4%) patients with grade I + II and 20 (90.1%) patients with grade III. Besides, there were 13 (50.0%) patients with positive HIF-1α expression and 50 (86.2%) patients with positive HIF-1α expression.

**Table 5:** The correlation between HIF-1α expression and clinical characteristics.

Variables	Group	Number	HIF-1α		χ^2^	*P*
			Positive	Negative		
Age	<45	30	20 (66.7%)	10 (33.3%)	1.728	.189
	≥45	54	43 (79.6%)	11 (20.4%)		
Tumor size	<2 cm	42	30 (71.2%)	12 (28.8%)	0.571	.450
	≥2 cm	42	33 (78.6%)	9 (21.4%)		
Vascular tumor thrombus	No	36	26 (72.2%)	10 (27.8%)	0.259	.611
	Yes	48	37 (77.1%)	11 (22.9%)		
Histological grade	I + II	62	43 (69.4%)	19 (20.6%)	4.032	.045
	III	22	20 (90.1%)	2 (9.9%)		
Ki-67	<20%	24	15 (62.5%)	9 (37.5%)	2.800	.094
	≥20%	60	48 (80.0%)	12 (20.0%)		
Lymph node metastasis	No	26	13 (50.0%)	13 (50.0%)	12.552	.000
	**Yes**	**58**	**50 (86.2%**)	**8 (13.8%**)		

### 3.6. The expression of CD147 in breast cancer

**Figure 4.**
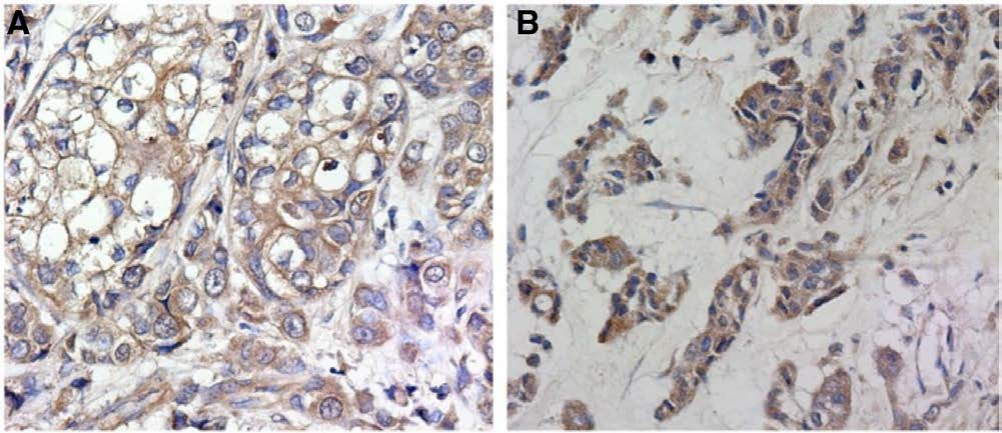
The positive expression characteristics of CD147 in the TNBC and fibroadenoma of the breast. (A) The expression of CD147 in TNBC. (B) The expression of CD147 in fibroadenoma of the breast. TNBC = triple-negative breast cancer.

**Figure 5.**
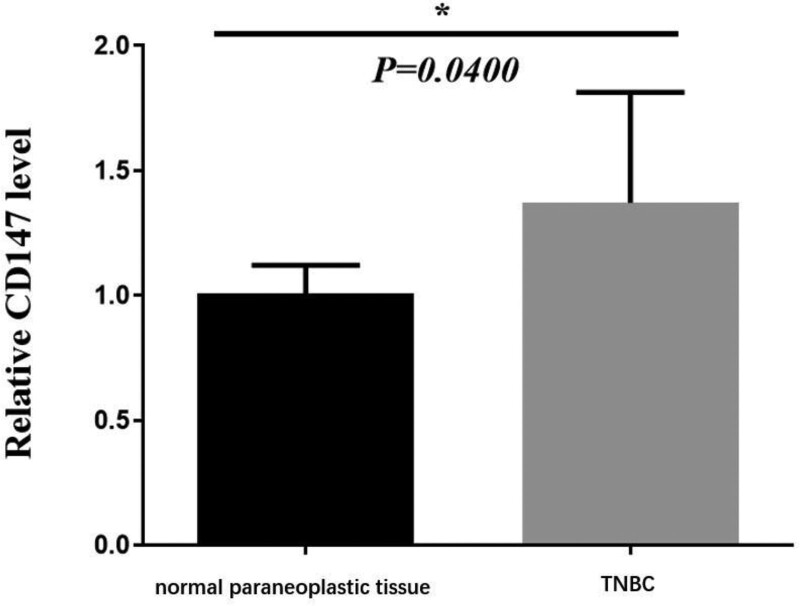
The expression of CD147 in TNBC and normal paraneoplastic tissue. *P < .05. TNBC = triple-negative breast cancer.

Additionally, we also identified the expression of HIF-1α in different types of breast cancer. As shown in Table 6, there was a significant difference of CD147 expression between TNBC and fibroadenoma of breast (67 [79.8%] vs 10 [25.0%], *P* < .001). The immunohistochemical results showed that HIF-1α was usually expressed in the cell membrane and cytoplasm, which was brownish yellow particles. (Fig. 4A-B). Furthermore, the results of qPCR showed that CD147 expression was upregulated in TNBC in comparison with normal samples (Fig. 5, *P* < .05).

**Table 6:** The expression of CD147 in TNBC and fibroadenoma of the breast.

Group	Number	CD147 positive expression	χ2	*P*
TNBC samples	84	67 (79.8%)	34.545	<.001
Fibroadenoma of breast samples	40	10 (25.0%)		

TNBC = triple-negative breast cancer.

### 3.7. The correlation between CD147 expression and clinical characteristics in triple-negative breast cancer

Furthermore, we determined the correlation between CD147 expression and clinical characteristics (Table 7). The results exhibited that the number of patients with positive CD147 expression in grade I + II was significantly more than that of grade III (46 [74.2%] vs 21 [95.5%], *P* < .05). The patients with Ki-67 value ≥ 20% have a high ratio of positive CD147 expression compared with the patients with Ki-67 < 20%. Besides, CD147 was positively expressed in 48 (82.8%) patients with lymph node metastasis and 19 (73.1%) patients without lymph node metastasis (*P* < .05).

**Table 7:** The correlation between CD147 expression and clinical characteristics.

Variables	Group	Number	CD147		χ^2^	*P*
			Positive	Negative		
Age	<45	30	21 (70.0%)	9 (30.0%)	2.775	.097
	≥45	54	46 (85.2%)	8 (14.8%)		
Tumor size	<2 cm	42	31 (73.8%)	11 (26.2%)	1.844	.175
	≥2 cm	42	36 (85.7%)	6 (14.3%)		
Vascular tumor thrombus	No	36	29 (80.6%)	7 (19.4%)	0.025	.875
	Yes	48	38 (79.2%)	10 (20.8%)		
Histological grade	I + II	62	46 (74.2%)	16 (25.8%)	4.547	.033
	III	22	21 (95.5%)	1 (4.5%)		
Ki-67	<20%	24	14 (58.3%)	10 (41.7%)	9.558	.002
	≥20%	60	53 (88.3%)	7 (11.7%)		
Lymph node metastasis	No	26	19 (73.1%)	7 (26.9%)	1.042	.037
	Yes	58	48 (82.8%)	10 (17.2%)		

### 3.8. The correlation between hypoxia-inducible factor-1alpha and CD147 in triple-negative breast cancer

**Figure 6.**
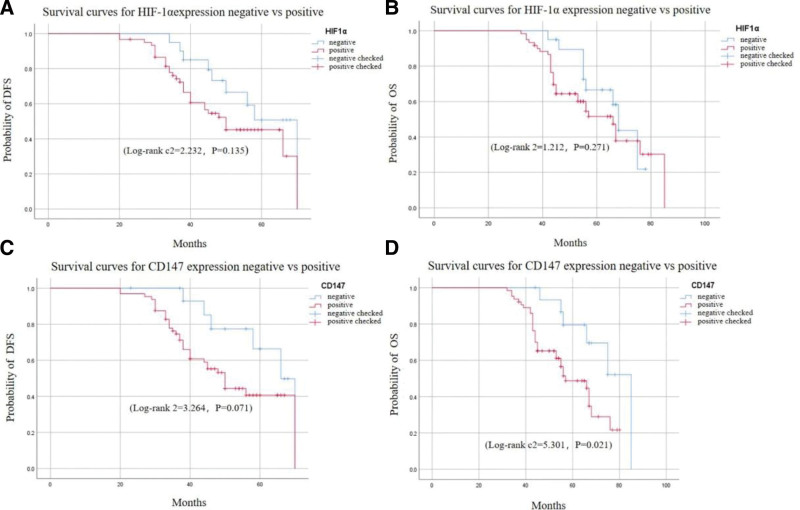
The prognosis value of HIF-1α and CD147 in TNBC. (A) K–M curve of HIF-1α in DFS of TNBC patients (B) K–M curve of HIF-1α in OS of TNBC patients (C) K–M curve of CD147 in DFS of TNBC patients (D) K–M curve of CD147 in OS of TNBC patients. DFS = disease free survival, OS = overall survival, TNBC = triple-negative breast cancer.

The further analysis showed that there was a significant relationship between the expression of HIF-1α and CD147 (Table 8, *P* < .001). Moreover, the survival analysis showed that HIF-1α expression was not related to the prognosis of TNBC (Fig. 6A-B), while CD147 was only significantly related to the OS in TNBC patients (Fig. 6D, *P* < .05 and Fig. 6C, *P* > .05).

**Table 8:** The correlation between HIF-1α and CD147 in TNBC.

HIF-1α	CD147		*r*	*P*
	Negative	Positive		
Negative	10	11	0.393	.000
Positive	7	56		

HIF-1α = hypoxia-inducible factor-1alpha.

## 4. Discussion

TNBC is a highly invasive malignant tumor, and its prognosis is worse than other subtypes of breast cancer.^[15]^ Although TNBC is sensitive to chemotherapy, patients have limited benefit from it.^[16]^ The main reason is that the tumor has strong drug resistance and obvious side effects of chemotherapy, and it is easy to relapse and metastasize.^[17,18]^ At present, chemotherapy is still its main adjuvant treatment. How to improve the prognosis of TNBC patients, reduce their recurrence and metastasis rate and transform them into chronic diseases is the focus of current research. Therefore, finding new effective therapeutic targets or molecular markers is the most important thing to improve the prognosis of TNBC patients.

Yang et al found that the positive expression rate of HIF-1α in TNBC tissue was 45.3%. The expression of HIF-1 was significantly correlated with the patient’s age, histological grade and lymph node status, but not with the expression of Ki-67. The multivariate Cox regression analysis showed that HIF-1α expression, histological grade and lymph node status were independent risk factors for postoperative survival of TNBC patients.^[19]^ Besides, Ge et al claimed that the positive expression rate of HIF-1α in TNBC tissue was 86.7%, which was significantly higher than that in its adjacent tissues (15.0%, 6/40). The expression of HIF-1α was related to the histological grade of TNBC and whether lymph nodes occurred or not, but not related to the patient’s age, tumor size, and menopause.^[20]^ Zhang et al demonstrated that the positive expression rate of HIF-1α in 103 patients with TNBC was 54.4%, and the expression of HIF-1α was significantly correlated with patients’ age, tumor size, histological grade, lymph node metastasis, and tumor stage. Kaplan–Meier survival curve and Log-rank analysis showed that HIF-1α can affect the 3-year disease-free survival of TNBC patients. The multivariate Cox regression analysis showed that HIF-1α expression was related to the 3-year disease-free survival of TNBC patients, but its value as an independent risk factor for prognosis was not found.^[21]^ In this study, the expression of HIF-1α in 84 cases of TNBC and 40 cases of breast fibroadenoma was detected by immunohistochemical technique. The positive rate of HIF-1α in the TNBC group was 75%, while that in the control group was 27.5%. The expression of HIF-1α in TNBC was significantly higher than that in the breast. In exploring the relationship between the expression of HIF-1α in TNBC tissue and the different clinicopathological factors of patients, we found that the expression of HIF-1α was related to the histological grade and lymph node metastasis of TNBC, but had no significant correlation with the patient’s age, tumor size, vascular tumor thrombus and Ki-67 expression, which was basically consistent with previous studies. In the survival analysis, we found that the DFS and OS time of the HIF-1α negative group was higher than the HIF-1α positive group, but there was no statistical difference between the 2 groups. The multivariate Cox regression analysis showed that the expression of HIF-1α could not be used as an independent prognostic factor for TNBC patients. The survival analysis curves (OS, DFS) based on the bioinformation also suggested that the expression of HIF-1α was not correlated with the prognosis of TNBC.

In the research of Wang et al, the positive expression rate of CDl47 in TNBC tissues was 81.82% and was significantly correlated with high histological grade, high expression of Ki-67 and positive expression of p53. The results of the survival analysis showed that the different expression intensity of CDl47 in TNBC tissue was related to the OS and DFS time. Multivariate Cox regression analysis showed that CD147 and Ki-67 were risk factors for the prognosis of TNBC. The higher the positive expression rate, the shorter the survival time of patients.^[22]^ Zhao et al found that expression of CD147 was observed in all 147 TNBC samples and was significantly correlated with histological grade, tumor size, Ki-67 and lymph node metastasis in TNBC. The study also found that CD147 was closely related to the PFS and OS of TNBC.^[23]^ In this study, CD147 was positively expressed in 79.8% of TNBC tissues. The positive rate of CD147 in the control group was 25.0%, which indicated that the expression of CD147 in the TNBC group was significantly higher than that in breast fibroadenoma tissue, which was consistent with previous studies. In exploring the relationship between the expression of CD147 in TNBC tissue and different clinicopathological factors of patients, we found that the expression of CD147 was related to histological grade, Ki-67 and lymph node metastasis, but not to the patient’s age, tumor size, and vascular tumor thrombus, which was basically consistent with previous studies. In the survival analysis, we found that the OS of the CD147 negative group was significantly better than the HIF-1α positive group.

In addition, we also found that the there was a close relationship between HIF1α and CD147 expression. A previous study revealed that the expression of HIF-1α was downregulated by CD147. Wang et al demonstrated that CD147 could induce angiogenesis through regulating HIF-1α expression.^[24]^ Thus, we suggested that CD147 could regulate the expression of HIF-1α to promote TNBC development through inducing angiogenesis. Although there were indeed some limitations of the study, such as limited sample sizes, single center, and retrospective study, we had already probed into the role of HIF-1a and CD147 in the prognosis of patients with TNBC, which might initiate noval thought for subsequent mechanism reseach.

In conclusion, we found that HIF-1α and CD147 were upregulated in TNBC, and CD147 was closely related to the OS of TNBC patients. Moreover, we found a close relationship between CD147 and HIF-1α, which suggested the potential mechanism of them in the development of TNBC.

## Author contributions

**Conceptualization:** Pai Peng, Chaohua Hu.

**Data curation:** Menghe Chen.

**Formal analysis:** Menghe Chen.

**Investigation:** Zitao Liu.

**Methodology:** Menghe Chen, Zitao Liu.

**Resources:** Menghe Chen, Zitao Liu, Kai Zheng.

**Software:** Kai Zheng, Chaohua Hu.

**Supervision:** Chaohua Hu.

**Validation:** Zitao Liu, Kai Zheng, Chaohua Hu.

**Visualization:** Kai Zheng.

**Writing – original draft:** Pai Peng.

**Writing – review & editing:** Pai Peng.

## References

[1] Zhong B Zhang J Wu J. Efficacy and safety of palbociclib plus endocrine therapy for patients with HR(+)/HER2(-) advanced breast cancer in real-world clinical practice. Ann Transl Med. 2022;10:362. 35434007 10.21037/atm-22-1002 PMC9011297

[2] Yang H Xue M Su P. RNF31 represses cell progression and immune evasion via YAP/PD-L1 suppression in triple negative breast Cancer. J Exp Clin Cancer Res. 2022;41:364. 36581998 10.1186/s13046-022-02576-y PMC9801641

[3] Zhao R Wei W Zhen L. WGCNA-based identification of potential targets and pathways in response to treatment in locally advanced breast cancer patients. Open Med (Wars). 2023;18:20230651. 36896338 10.1515/med-2023-0651 PMC9990777

[4] Sharma P Barlow WE Godwin AK. Validation of the DNA damage immune response signature in patients with triple-negative breast cancer from the SWOG 9313c trial. J Clin Oncol. 2019;37:3484–92. 31657982 10.1200/JCO.19.00693 PMC7194448

[5] Alsharabasy AM Glynn S Farras P Pandit A. Interactions between nitric oxide and hyaluronan implicate the migration of breast cancer cells. Biomacromolecules. 2022;23:3621–47. 35921128 10.1021/acs.biomac.2c00545 PMC9472231

[6] O’Reilly EA Gubbins L Sharma S. The fate of chemoresistance in triple negative breast cancer (TNBC). BBA Clin. 2015;3:257–75. 26676166 10.1016/j.bbacli.2015.03.003 PMC4661576

[7] Byun Y Choi YC Jeong Y. MiR-200c downregulates HIF-1alpha and inhibits migration of lung cancer cells. Cell Mol Biol Lett. 2019;24:28. 31061665 10.1186/s11658-019-0152-2 PMC6487019

[8] Liu Q Guan C Liu C Li H Wu J Sun C. Targeting hypoxia-inducible factor-1alpha: a new strategy for triple-negative breast cancer therapy. Biomed Pharmacother. 2022;156:113861. 36228375 10.1016/j.biopha.2022.113861

[9] Xiang L Gilkes DM Hu H. HIF-1alpha and TAZ serve as reciprocal co-activators in human breast cancer cells. Oncotarget. 2015;6:11768–78. 26059435 10.18632/oncotarget.4190 PMC4494903

[10] Fourie C du Plessis M Mills J Engelbrecht AM. The effect of HIF-1alpha inhibition in breast cancer cells prior to doxorubicin treatment under conditions of normoxia and hypoxia. Exp Cell Res. 2022;419:113334. 36044939 10.1016/j.yexcr.2022.113334

[11] Seo YR Lee J Ryu HS. Lateral interactions between CD276 and CD147 are essential for stemness in breast cancer: a novel insight from proximal proteome analysis. Sci Rep. 2023;13:14242. 37648771 10.1038/s41598-023-41416-7 PMC10469185

[12] Kong LM Liao CG Zhang Y. A regulatory loop involving miR-22, Sp1, and c-Myc modulates CD147 expression in breast cancer invasion and metastasis. Cancer Res. 2014;74:3764–78. 24906624 10.1158/0008-5472.CAN-13-3555

[13] Xiong L Ding L Ning H. CD147 knockdown improves the antitumor efficacy of trastuzumab in HER2-positive breast cancer cells. Oncotarget. 2016;7:57737–51. 27363028 10.18632/oncotarget.10252 PMC5295386

[14] Toole BP Slomiany MG. Hyaluronan, CD44 and Emmprin: partners in cancer cell chemoresistance. Drug Resist Updat. 2008;11:110–21. 18490190 10.1016/j.drup.2008.04.002 PMC2584579

[15] Liu Q Song X Liu Z Yu Z. Investigation of candidate genes and pathways in Basal/TNBC patients by integrated analysis. Technol Cancer Res Treat. 2021;20:15330338211019506. 34184566 10.1177/15330338211019506 PMC8246569

[16] Rousseau C Ruellan AL Bernardeau K. Syndecan-1 antigen, a promising new target for triple-negative breast cancer immuno-PET and radioimmunotherapy. A preclinical study on MDA-MB-468 xenograft tumors. EJNMMI Res. 2011;1:20. 22214534 10.1186/2191-219X-1-20 PMC3250983

[17] Xie K Ren X Hong X. Platinum-based adjuvant therapy was efficient for triple-negative breast cancer: a meta-analysis from randomized controlled trials. Bioengineered. 2022;13:14827–39. 36278891 10.1080/21655979.2022.2115616 PMC9601551

[18] Guan X Gu S Yuan M Zheng X Wu J. MicroRNA-33a-5p overexpression sensitizes triple-negative breast cancer to doxorubicin by inhibiting eIF5A2 and epithelial-mesenchymal transition. Oncol Lett. 2019;18:5986–94. 31788073 10.3892/ol.2019.10984 PMC6865640

[19] Yang JG Yang H Wang L. Hypoxia-inducible factor 1α combined with c-myc predicts postoperative survival in triple-negative breast cancer patients. J Clin Surg. 2022;30:1172–5.

[20] Ge ZY, TY, Wang B. Expression and clinical value of SIRT6 and HIF-1α in triple-negative breast cancer. Med Inf. 2019;32:95–7.

[21] Zhang Y, GA, Zhang XJ. Clinical significance of PD-L1 and HIF-1α expression in triple-negative breast cancer tissues. Chin J Tumor Control. 2018;25:1303–7.

[22] Wang C Shan M Niu RJ. Expression of CD147 in triple-negative breast cancer and its relationship with prognosis. J Shanghai Jiao Tong Univ (Med Ed). 2017;37:55–9.

[23] Zhao S Ma W Zhang M. High expression of CD147 and MMP-9 is correlated with poor prognosis of triple-negative breast cancer (TNBC) patients. Med Oncol. 2013;30:335. 23263825 10.1007/s12032-012-0335-4 PMC4059361

[24] Wang CH Yao H Chen LN. CD147 induces angiogenesis through a vascular endothelial growth factor and hypoxia-inducible transcription factor 1alpha-mediated pathway in rheumatoid arthritis. Arthritis Rheum. 2012;64:1818–27. 22170561 10.1002/art.34341

